# The contribution of the Italian residents in neurology to the COVID-19 crisis: admirable generosity but neurological training remains their priority

**DOI:** 10.1007/s10072-021-05346-4

**Published:** 2021-08-10

**Authors:** Cristina Tassorelli, Vincenzo Silani, Alessandro Padovani, Paolo Barone, Paolo Calabresi, Paolo Girlanda, Leopnardo Lopiano, Luca Massacesi, Salvatore Monaco, Marco Onofrj, Gioacchino Tedeschi, Alfredo Berardelli

**Affiliations:** 1 Department of Brain and Behavioural Sciences, University of Pavia, Pavia, Italy; 2 IRCCS Mondino Foundation, Via Mondino 2, 27100 Pavia, Italy; 3 Department of Neurology-Stroke Unit and Laboratory Neuroscience, Istituto Auxologico Italiano, IRCCS, Milan, Italy; 4 Department of Pathophysiology and Transplantation “Dino Ferrari Center”, University of Milan, Milan, Italy; 5 Department of Clinical and Experimental Sciences, Neurology Unit, University of Brescia, Brescia, Italy; 6 Department of Medicine, Surgery and Dentistry, University of Salerno, Salerno, Italy; 7 Neurology Unit, Fondazione Policlinico Universitario A. Gemelli IRCCS, Rome, Italy; 8 Department of Neuroscience, Università Cattolica del Sacro Cuore, Rome, Italy; 9 Department of Clinical and Experimental Medicine, University of Messina, Messina, Italy; 10 Department of Neuroscience, University of Torino, Turin, Italy; 11 Department of Neurosciences, Drug and Child Health, University of Florence, Florence, Italy; 12 Department of Neurosciences, Biomedicine and Movement Sciences, University of Verona, Verona, Italy; 13 Department of Neuroscience, Imaging and Clinical Sciences “G. D’Annunzio”, University of Chieti, Chieti, Italy; 14 Department of Advanced Medical and Surgical Sciences, University of Campania “Luigi Vanvitelli”, Napoli, Italy; 15 Department of Human Neurosciences, Sapienza University of Rome, Rome, Italy; 16 IRCCS Neuromed Institute, Pozzilli, IS Italy

**Keywords:** COVID-19, Residents in neurology, Training, Real-world experience, Early employment

## Abstract

**Background:**

The coronavirus disease 2019 (COVID-19) pandemic has severely impacted the Italian healthcare system, underscoring a dramatic shortage of specialized doctors in many disciplines. The situation affected the activity of the residents in neurology, who were also offered the possibility of being formally hired before their training completion.

**Aims:**

(1) To showcase examples of clinical and research activity of residents in neurology during the COVID-19 pandemic in Italy and (2) to illustrate the point of view of Italian residents in neurology about the possibility of being hired before the completion of their residency program.

**Results:**

Real-life reports from several areas in Lombardia—one of the Italian regions more affected by COVID-19—show that residents in neurology gave an outstanding demonstration of generosity, collaboration, reliability, and adaptation to the changing environment, while continuing their clinical training and research activities. A very small minority of the residents participated in the dedicated selections for being hired before completion of their training program. The large majority of them prioritized their training over the option of earlier employment.

**Conclusions:**

Italian residents in neurology generously contributed to the healthcare management of the COVID-19 pandemic in many ways, while remaining determined to pursue their training. Neurology is a rapidly evolving clinical field due to continuous diagnostic and therapeutic progress. Stakeholders need to listen to the strong message conveyed by our residents in neurology and endeavor to provide them with the most adequate training, to ensure high quality of care and excellence in research in the future.

**Supplementary Information:**

The online version contains supplementary material available at 10.1007/s10072-021-05346-4.

## Introduction

The pandemic due to the coronavirus disease 2019 (COVID-19) has severely impacted the healthcare system in the world and in Italy for a sustained period of time [1–3]. Since March 2020, the majority of Italian hospitals have rapidly shifted their target and mission from non-urgent, specialized, or highly specialized care to urgent and non-urgent COVID-19 care. During the first wave of the pandemic, the impact hit mostly the North Italian Regions, but then the entire Italian territory during the second wave, which affected also regions relatively spared by the first wave.

In this setting, and considering the pre-existent limited availability of specialists, Italian hospitals found themselves in a critical shortage of doctors. The scary gap was partially filled with the support of residents in those hospitals that delivered training for residents in the different medical programs. For most of the residents, the choice to help COVID-19-dedicated wards entailed the interruption of their discipline-specific training, which was anyway made inescapable by the generalized reconversion of wards dedicated to their discipline into COVID-dedicated structures.

The Italian government sought to facilitate and formalize the contribution of residents to the management of the COVID-19 crisis by releasing an urgent decree aimed at potentiating the National Health System. Basically, the decree allowed hospitals to hire residents in their 2 last years of training program (DECRETO-LEGGE 9 marzo 2020, “Disposizioni urgenti per il potenziamento del Servizio Sanitario Nazionale in relazione all’emergenza COVID-19”). In this setting, the classic training in neurology, as in many other disciplines, was deeply challenged.

In this paper, we illustrate representative real-world experiences showing the practical reaction of residents in university hospitals hosting a residency program in Lombardia, the Italian region most affected by both waves of COVID-19, with its more than 500,000 cases at the time of writing. Furthermore, we present the data of a survey conducted among the Italian residents in neurology to gather their point of view about the possibility of being formally hired according to the governmental decree.

## Real-world experiences in Northern Italy

### Pavia

The neurology residency program at the University of Pavia has its official clinical site at the National Neurological Research Institute C. Mondino Foundation, a standing alone neurological hospital with more than 130 beds distributed in units specialized in different neurological fields: movement disorders, multiple sclerosis, headaches, stroke and emergency neurology, neuroinfectivology, general neurology, neurorehabilitation, and child neuropsychiatry. The training also includes rotations at the stroke unit located in the General Hospital of Pavia, Policlinico San Matteo.

During the first COVID-19 wave, the rotation of residents in the different units of the program network were severely impacted due to the internal re-organization at the Mondino Institute and because of the increased demand of support from the stroke unit located in the General Hospital. More residents were dislocated to this latter unit. At the same time, more residents were called to support the neurorehabilitation unit of the Mondino Foundation, one of the few neurorehabilitation units in Lombardy that remained COVID-19-free and was therefore overwhelmed by requests of admission of COVID-19-negative neurological patients in need of rehabilitation from different hospitals of the Lombardia Region.

Soon, the Mondino governance realized the need to open a unit dedicated to the acute care of neurological patients who tested positive for COVID-19 (with/without neurological manifestations of coronavirus disease) to accommodate their specific care requirements, and thus, the residents’ rotations were adapted to also cover the said unit.

The situation was quite similar during the second wave, with the difference that the COVID unit at the Mondino Institute was immediately re-activated and some additional beds were dedicated to COVID-free acute stroke patients.

In this year lived with COVID, all the Pavia neurology residents have given an outstanding demonstration of generosity, collaboration, reliability, and adaptation to the changing environment. Probably as the consequence of their dedication to work and the huge effort sustained by their tutors to continue the practical training and to deliver remote theoretical teaching, all the residents have passed their annual evaluation last November. They also managed to continue their research activities and to expand them to include COVID-related issues [4–7].

None of the Pavia residents in neurology has applied for the hiring procedures in other hospitals according to the governmental decree.

### Milan

The University of Milan Medical School provides the second largest residency program in Italy articulated with seven different teaching hospitals. The institutions have been differently affected by the reconversion of wards into COVID-dedicated structures and some of them have been completely reconverted to dedicated COVID-19 wards in the first and second pandemic waves. The second wave reproduced the same situation. As a consequence, several neurology residents were recruited or volunteered for COVID-19-dedicated wards.

Overall, the COVID-19 pandemic had a negative effect on the formation as neurologists concerning didactics, clinical, and research training. Training abroad has been canceled or postponed. Virtual and remote learning have been implemented and different surveys demonstrated the concern to ensure continued quality of trainees’ neurologic education [7]. Neurology residents also largely contributed in describing the neurological aspects of COVID-19 with original studies [8, 9]. Some of the residents in neurology applied for the hiring in other hospitals according to the governmental decree.

All the Milano neurology residents have given an outstanding demonstration of collaboration providing an excellent standard of care even in the non-neurological wards. All the residents have passed their annual evaluation on November 2020 with excellent marks.

### Brescia

The neurology residency program at the University of Brescia has its official clinical site at the ASST Spedali Civili of Brescia, a teaching hospital with more than 1000 beds including 40 beds of general neurology, 10 beds of vascular neurology and stroke unit, and 4 beds of day hospital. Additionally, there is an out-patient-dedicated neuropathophysiology unit (EEG, EMG/ENG, EPs, Epilepsy Centre, Sleep Centre). The neurology units include an out-patient service for movement disorders, neuroimmunology, headaches, stroke and emergency neurology, neuroinfectivology, neurodegenerative disorders, neuromuscular diseases, and neuropsychology. The training also includes rotations at the neurology units of three different hospitals such as Poliambulanza Hospital (Brescia), ASST Franciacorta (Chiari), and ASST Cremona. Furthermore, the school is committed with two rehabilitation centers (Centro Don Gnocchi di Rovato and FERB Trescore Balneario). The education program included formal lessons, study groups, journal clubs, and clinical rounds and all are involved in active scientific activity.

At the beginning of the COVID-19 outbreak, the rotation of residents in the different units of the program network was interrupted according to the university indication. Most of them were initially dedicated to outpatients, but this activity was also soon interrupted. On March 8th, a new rotation schedule was established, which limited the residents program to the neurology units and the emergency room H24 of the Teaching Hospital of Brescia. By March 24th, a 16-bed NeuroCOVID Unit was established in the teaching hospital in Brescia and residents were called to support its activity H24. At this point, rotation of the residents included the General Neurology Unit, the Vascular Neurology and Stroke Unit, the Neurophysiopathology Unit, and the NeuroCOVID Unit. Residents were also on call during the night shifts for all the services and tutored by the H24 senior neurologists. The other neurology units of the school education network were closed for the entire first-wave lockdown. By the end of May, the NeuroCOVID Unit closed while the other neurology units reopened and the residents started to rotate again within the entire network. In October 2021, when the second wave started, the NeuroCOVID Unit was rapidly reactivated. This time, residents maintained the ordinary rotation regimen whereas only three residents voluntarily asked to work in the NeuroCOVID Unit, supporting data entry, clinical assistance, and research studies. Noteworthy, all residents were involved in different research activities on neuroCOVID-19 patients and contributed to many scientific papers [9–15], while continuing their theoretical education by means of virtual training (journal clubs, clinical rounds, and academic lectures). All residents went through and passed the annual examination according to planned schedule, and they were actively involved in the “End-of-Year Highlights” session presenting their scientific progresses and programs.

Only one resident (attending the 4th year of the school) applied with success for the hiring procedure and since November is now part of the staff of the NeuroCOVID Unit.

## The national survey to gather the feedback from residents

In June 2020, a questionnaire was created by the authors with the help of a group of residents and was circulated by e-mail to the Italian residents in neurology attending the 35 neurology residency programs of the Italian Universities.

The questionnaire was formed by two sections: the first one was aimed at profiling the residents (information about the region where their school was set and their year into residency); the second one was formed by three questions with associated sub-questions (supplementary information 1). The questionnaire inquired about the participation in the selection for hire dedicated to residents in neurology according to the decree, and investigated the reasons for participating or for abstaining, respectively. Those residents who participated in the selection for hire were asked about their experience. Those who abstained from participating were asked whether they would be willing to participate in future competitions.

Participation to the survey was free. Replies received by e-mail were collected by a person who was not involved in the study and in data analysis (CT Secretary). This person was instructed to populate a fully anonymized excel datasheet with the answers to questions that are evaluated in this paper.

With regard to ethical issues, given the independent nature of the survey, the measures taken to ensure anonymity, and the absence of sensitive patients’ data in the questionnaire, and in accordance with Italian regulations, no formal approval of the survey was required from the Institutional Review Boards of the participating centers.

## Results

A total of 549 questionnaires from 30 out of the 35 neurology residency programs were returned. Of these, 257 were from the residents in their 3rd and 4th year of training and are presented here, in consideration of the fact that only the 3rd and 4th year were granted the option to participate in the dedicated selection for hire according to the national decree.

*Anwers to question 1*

*Did you participate in the selection-for-hire dedicated to Residents in Neurology, according to the decree of March 9, 2020, aimed at hiring doctors for the care of patients during the COVID-19 crisis?*

Only 14 residents answered “yes” to this question, and 201 replied negatively. For the remaining 42 residents, the question was “not applicable” because no selection-for-hire was available in the regions where their program was located. The geographic distribution of the residents who participated in the dedicated competitions were as follows: Lombardia (n 4), Emilia Romagna (n 4), Campania (n 2), Lazio (n 2), Marche (n 1).

The reasons for participating were as follows: “Desire to actively contribute with my professional support during the health emergency” for 13 respondents. One of them also indicated “Economic opportunity,” and another “Training opportunity.” The answer was missing for the remaining residents.

Only 4 out of the 14 residents who participated in the selection for hire were actually hired; all of whom were employed in team management of patients and felt adequately trained for the duties they were assigned to. Only 2 of them reported that they received some training from the staff of the hospital where they were hired.

**Fig. 1:**
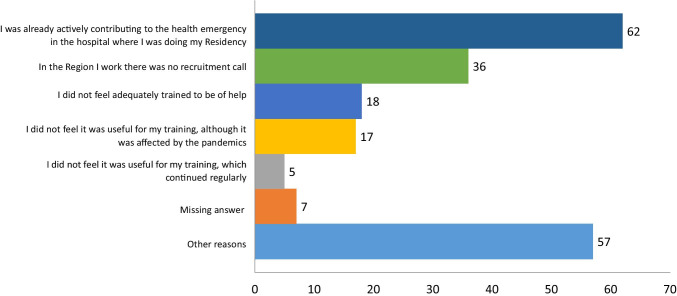
Reasons for not participating in the selection-for-hire dedicated to the residents in neurology, according to the Decree of March 9, 2020. Data are presented as absolute number. One subject ticked two options

For those 201 residents who did not participate in the selection for hire dedicated to residents in neurology, the reasons behind their decision are illustrated in Fig. 1.

*Answers to question 2*

*Once this health emergency is over, would you be willing to participate in a competition dedicated to residents in a hospital within the training network or your residency program?*

**Fig. 2:**
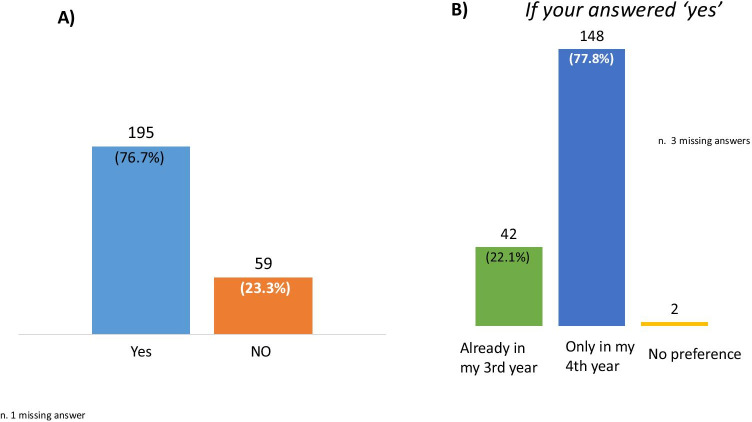
**A**) Answers to the question “*Once this health emergency is over, would you be willing to participate in a selection-for-hire reserved to Residents in Neurology in a hospital within the training network of your Residency program?*” **B**) The bars show the preferred time to participate in a selection-for-hire dedicated to residents in neurology in a hospital within the training network of the residency program for those who answered “yes” in **A**. Data are presented as absolute number (%). One subjects ticked two options. One missing answer in **A** and 3 missing answers in **B**

The large majority of residents answered “yes” to this question: 195 out of 254 (76.7%), but still they preferred being hired in their 4th year of residency (Fig. 2).

*Answers to question 3*

*Once this health emergency is over, would you be willing to participate in a competition dedicated to residents in a hospital outside the training network or your residency program?*

The majority of residents answered “yes” to the question in a percentage that was slightly lower as compared to question 2: 159 out of 254 (62.6%). In this case, the preference regarding the year into training was less informative as we counted 111 missing answers.

## Discussion

The data obtained from the real-world experience in representative neurology residency programs in Italy and from the answers to the survey show that a relevant number of residents in neurology generously offered their professional support during the COVID-19 pandemic in the hospitals where they were being trained. This evidence is in keeping with the output of the survey conducted among the residents, which confirms their willingness to give their contribution to the health crisis. The results of the survey also clearly underscore the determination of the residents to complete their training in the best possible way. This is suggested by the very low number of residents who participated in the dedicated selections for hire and by the motivations provided for such a decision. The very large majority of residents were favorable to being hired in the hospital where they were performing training, but they preferred to do it mostly in their 4th year, once again prioritizing training over early employment.

The residency in neurology, whose duration in Italy was 5 years until 2015, has then been reduced to 4 years due to a political decision whose motivations are beyond the scope of this manuscript. A recent analysis of the European residency programs in neurology [16] revealed the lack of harmonization as regards the duration of the neurology residency programs across Europe, and only a minority of countries adopted the 48-month duration like Italy. On the contrary, in most of the European countries, the duration of the neurology residency programs is 60 months. Such a duration is consistent with the recommendations of the European Union of Medical Specialists (**UEMS**, Union Européenne des Médecins Spécialistes), section of Neurology [17], which require a minimum of 4 years of core neurology training, plus additional months for external medical disciplines. Another issue that requires attention is the final examination, an important element of the residency training program. Harmonization of a European examination has been initiated via the European Board of Examination organized by the UEMS, Section of Neurology (https://www.uems-neuroboard.org/web/index.php/european-board-examination). However, in Italy, this examination is not considered equivalent to the national certification, even if it has been recommended by several directors of Italian residency programs. A critical reevaluation of the structure of the neurology residency program has been proposed [18]. This foresees that the first 2 years of the program are mostly devoted to basic neurology in the in-patient and out-patient services of emergency, intensive care, and neurological units. The second part of the residency program should instead be more focused on neurological subspecialities, e.g., stroke, movement disorders, and neurorehabilitation (European Training Requirements for Neurology, version 2020, submitted).

Neurology is rapidly evolving as a result of continuous diagnostic and therapeutic progress, which influences the daily work of the neurologist. COVID-19 represents a striking example of the required continuous flexibility of modern neurology. Targeted actions need to be put in place by stakeholders to deliver the most adequate training to residents in neurology, to ensure quality of care and excellence in research.

The increasing knowledge about the impact of COVID-19 on the nervous system and the growing awareness that new global health crises that may occur in the future impose the need to expand the interest of neurologists and require a critical reevaluation of the structure of the neurology residency program in order to achieve the necessary blend between management of emergency conditions and expertise in neurological subspecialities [19].

## Supplementary Information

Below is the link to the electronic supplementary material.

Supplementary file1 (DOCX 16 KB)
