# Supplementary material for: The contribution of the Italian residents in neurology to the COVID-19 crisis: admirable generosity but neurological training remains their priority
Source: Neurol Sci. 2021 Aug 10;42(11):4425–31. doi: 10.1007/s10072-021-05346-4 (PMC8353936; doi:10.1007/s10072-021-05346-4)
Supplement: Supplementary file 1 — Supplementary file1 (DOCX 16 KB) [file 10072_2021_5346_MOESM1_ESM.docx]

**Supplementary material 1 - Questionnaire used for the Survey**

Profiling information

- Please indicate the Italian Region where you Residency Program is located:
- Please indicate the years in your training

1. First or Second
2. Third or Fourth

**Question 1**

*Did you participate to a recruitment competition dedicated to Residents in Neurology, according to the decree of March 9 2020, aimed at hiring doctors for the care of patients during the COVID-19 crisis?*

1. Yes
2. No

*If you answered ‘Yes’ to Q1,*

*Q1a) Why did you decide to participate?*

1. Desire to actively contribute with my professional support during the health emergency
2. Economic opportunity
3. Training opportunity
4. Other reasons

*Q1b) Were you actually hired?*

1. Yes
2. No

If you answered ‘yes’ to Q1b,

*Q1b1) To which duties were you assigned?*

1. Independent management of patients
2. Team management of patients
3. Administrative tasks
4. Other duties

*Q1b2) Did you feel adequately trained for the duties you were entrusted with?*

1. Yes
2. No

*Q1b3) Did you receive any training from the staff of the hospital that hired you?*

1. Yes
2. No

*Q1b4) Would you make this choice again in the future, if requested?*

1. Yes
2. No

*If you answered ‘No’ to Q1,*

*Q1c1) What was the reason?*

1. I was already actively providing my professional contribution to the health emergency in the hospital where I was doing my Residency
2. I did not feel adequately trained
3. I did not feel the choice useful for my training, which continued regularly
4. I did not feel the choice useful for my training, although this was affected by the pandemics
5. In the Region I work there were no dedicated recruitment competitions
6. Other reasons

**Question 2**

*Once this health emergency is over, would you be willing to participate in a competition dedicated to Residents in a hospital within the training network or your Residency program?*

1. *Yes*
2. *No*

*If you answered ‘Yes’ to Q2, please specify*

1. Already from my third year of Residency training
2. Only in the last year (4^th^) of Residency training

**Question 3**

*Once this health emergency is over, would you be willing to participate in a competition dedicated to Residents in a hospital outside of the training network or your Residency program?*

1. *Yes*
2. *No*

*If you answered ‘Yes’ to Q3, please specify*

1. Already from my third year of Residency training
2. Only in the last year (4^th^) of Residency training
